# A systematic scoping review evaluating sugar-sweetened beverage taxation from a systems perspective

**DOI:** 10.1038/s43016-023-00856-0

**Published:** 2023-10-19

**Authors:** Miriam Alvarado, Jean Adams, Tarra Penney, Madhuvanti M. Murphy, Safura Abdool Karim, Nat Egan, Nina Trivedy Rogers, Lauren Carters-White, Martin White

**Affiliations:** 1 MRC Epidemiology Unit, University of Cambridge School of Clinical Medicine, Institute of Metabolic Science, Cambridge Biomedical Campus, Cambridge, UK; 2 Global Food System and Policy Research, School of Global Health, Faculty of Health, York University, Toronto, Ontario Canada; 3 George Alleyne Chronic Disease Research Centre, Caribbean Institute for Health Research, The University of the West Indies, Bridgetown, Barbados; 4 Berman Institute, Johns Hopkins University, Baltimore, MD USA; 5 SPECTRUM Consortium, Usher Institute of Population Health Sciences and Informatics, Old Medical School, University of Edinburgh, Edinburgh, UK

**Keywords:** Risk factors, Epidemiology, Complex networks, Social policy

## Abstract

Systems thinking can reveal surprising, counterintuitive or unintended reactions to population health interventions (PHIs), yet this lens has rarely been applied to sugar-sweetened beverage (SSB) taxation. Using a systematic scoping review approach, we identified 329 papers concerning SSB taxation, of which 45 considered influences and impacts of SSB taxation jointly, involving methodological approaches that may prove promising for operationalizing a systems informed approach to PHI evaluation. Influences and impacts concerning SSB taxation may be cyclically linked, and studies that consider both enable us to identify implications beyond a predicted linear effect. Only three studies explicitly used systems thinking informed methods. Finally, we developed an illustrative, feedback-oriented conceptual framework, emphasizing the processes that could result in an SSB tax being increased, maintained, eroded or repealed over time. Such a framework could be used to synthesize evidence from non-systems informed evaluations, leading to novel research questions and further policy development.

## Abstract (web summary)

An illustrative, feedback-oriented conceptual framework is presented, emphasizing the processes that could result in a sugar-sweetened beverage tax being increased, maintained, eroded or repealed over time.

## Main

Population health interventions (PHIs) are typically policy- or infrastructure-related actions delivered at scale, often with an emphasis on disease prevention^1^, that aim to change contexts (for example, social, fiscal or physical environments) to reduce risk factors. Sugar-sweetened beverage (SSB) taxes are a PHI intended to reduce SSB consumption by both dampening consumer demand and encouraging industry-led reformulation. SSB taxes also provide governments with additional revenue, while potentially reducing future healthcare costs^2^. High levels of SSB consumption can harm health^3^ and the World Health Organization recommends SSB taxes as part of a broader strategy to reduce the burden of non-communicable diseases^2,4^.

SSB taxes have been implemented around the world, with over 70 jurisdictions introducing some form of SSB taxation since 2010^5^. Reviews of observational quantitative studies have indicated the effectiveness of SSB taxes at increasing SSB prices, reducing SSB sales and prompting reformulation^6–8^. Reviews of modelling studies have suggested that SSB taxes reduce premature mortality, increase government revenue and reduce expenditures over time^9^. Reviews of the policy process have highlighted key factors that have enabled or impeded the design and implementation of SSB taxation^10,11^. However, most of these reviews have considered evidence regarding the processes leading to SSB tax introduction separately from evidence regarding the impacts of SSB taxation, yet the interplay between these factors is critically important^12^. A systems thinking approach can help to further bring these perspectives together^13^.

A systems thinking approach may also reveal surprising, counterintuitive or unintended reactions to SSB taxation, including instances of potential policy resistance. Policy resistance entails a special class of unintended consequences that diminish the intended goal of a policy as actors within the system adapt to the policy change in unpredicted ways (Supplementary Text 1)^13^. For example, consumers may respond to the introduction of a tax by purchasing lower-cost SSBs (‘brand down switching’)^14^ or purchasing drinks in untaxed neighbouring jurisdictions^15^, manufacturers may introduce new low-cost SSBs^16^ and distributors may increase prices strategically and unevenly between products and across localities^17–19^. Not all unintended consequences are negative; some may support the original aims of a policy. For example, the introduction of an SSB tax itself may inadvertently convey health risk information to consumers and influence social norms, producing an unintended policy-supporting effect^20^. Using a feedback-oriented conceptual model could help to identify effective leverage points and pre-empt potential policy resistance, leading to greater and more sustained impact over time^21,22^.

Here we explored peer-reviewed publications on SSB taxation from a systems informed, feedback-oriented perspective to explore influences and impacts assessed in evaluation studies and to broadly characterize the types of methods used. We considered a subset of studies that looked at both influences and impacts and those studies that were explicitly systems informed, describing the methods used and types of insight generated in both cases. Finally, we developed a feedback-informed conceptual framework that ‘closes the loops’ between influences and impacts, that could inform future evaluations and synthesize multiple kinds of evidence concerning SSB taxation.

## Results

**Fig. 1:**
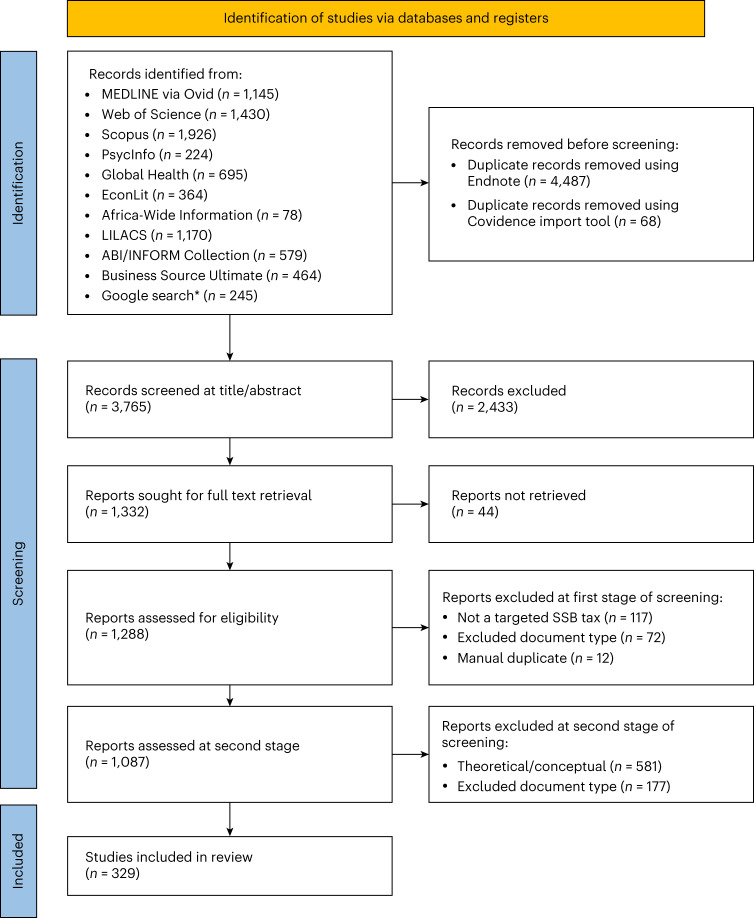
Preferred Reporting Items for Systematic Reviews and Meta-Analyses extension for Scoping Reviews flow diagram. The figure summarizes the search strategy and inclusion/exclusion chart associated with our systematic scoping review concerning SSB taxation. Multiple Google searches resulted in duplicate records that were not re-downloaded by hand in each search, resulting in the reported Google search total being less than 300. The figure is based on guidance from ref. ^79^.

We screened 3,765 studies and identified 1,087 studies for inclusion at stage 1 and 329 studies for inclusion at stage 2 (Fig. 1). All studies included at stage 2 and associated characteristics are summarized in ref. ^23^.

**Fig. 2:**
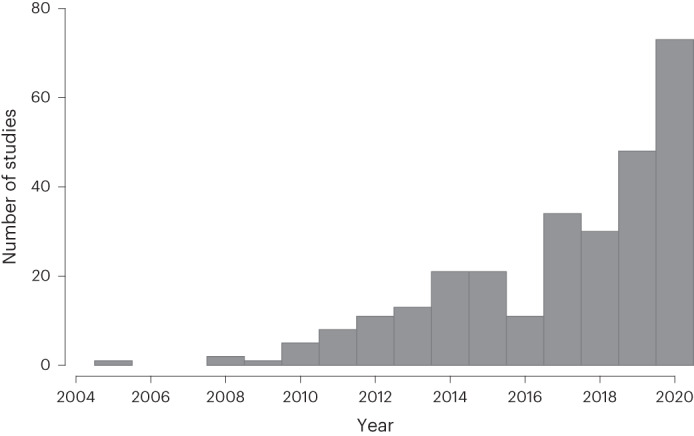
Number of studies on SSB taxation over time. The figure summarizes the frequency of papers on SSB taxation by year of publication based on our systematic scoping review. See Source Data Fig. 3 for a list of references of the 329 records included in the analysis.

The number of original academic analyses of SSB taxation have increased substantially over time, with 21 studies published in both 2014 and 2015, 48 in 2019 and 73 in 2020 (Fig. 2). This mirrors the increase in number of SSB taxes implemented over the same period^5^.

The largest number of studies focused on the United States, followed by the United Kingdom, Mexico, South Africa and Australia.

### Influences and impacts related to SSB taxation

We extracted an initial list of 152 potential influences and impacts based on the reviews and conceptual papers selected from the first stage of screening. We identified an additional 476 influence/impact factors by reviewing the included empirical papers for a total of 628 potential influences and impacts. We clustered conceptually similar influences/impacts into parent factors, developing a final list of 57 factors (9 influences and 48 impacts). Supplementary Table 1 includes a full list and detailed definition of each parent factor.

**Fig. 3:**
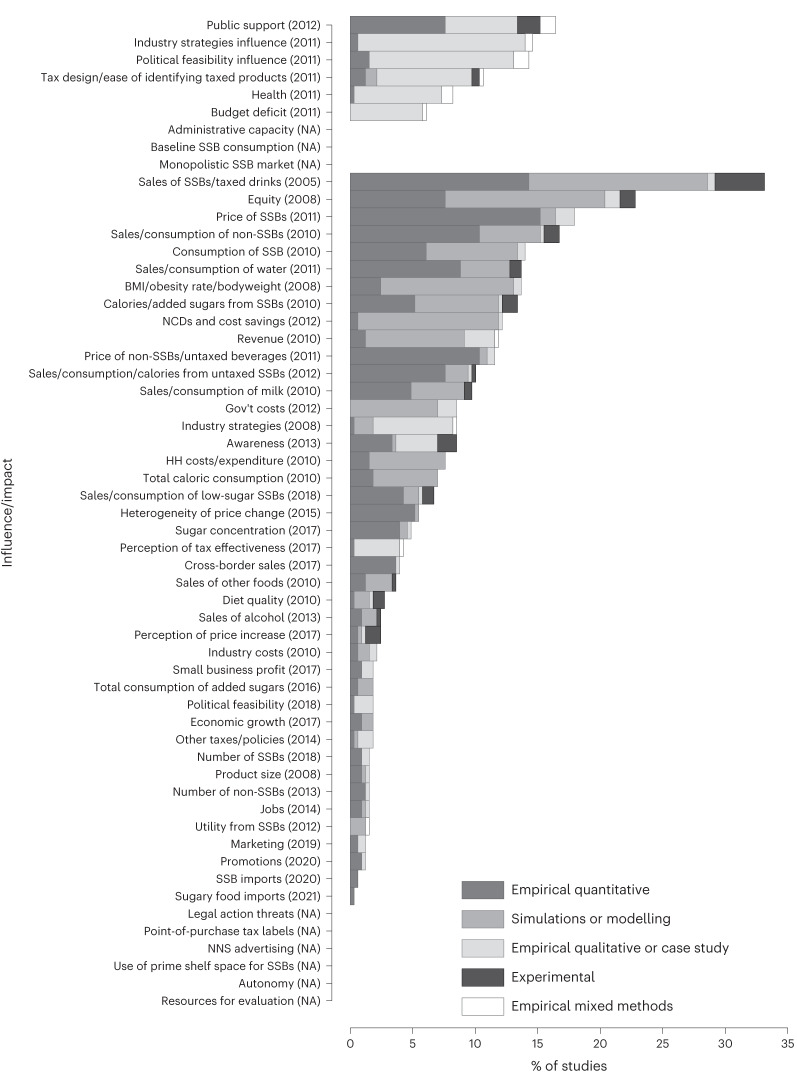
Percentage of studies that assessed identified influences or impacts of SSB taxation. The figure summarizes the percentage of the 329 studies included in the systematic scoping review that assessed any of 57 identified influences or impacts concerning SSB taxation. We report the year each influence/impact was first assessed in parentheses and display the frequency with which each type of study (empirical quantitative, simulations or modelling, empirical qualitative or case study, experimental, empirical mixed methods) was used to assess each influence/impact. NA, not applicable; BMI, body mass index; NCDs, non-communicable diseases; Gov't, government; hh, household; NNS, non-nutritive sweeteners. Source data

Of all studies identified, 111 studies assessed at least one influence (34%). The most-studied influences were public support and industry strategies. Most influences were primarily assessed using qualitative methods except for public support (Fig. 3 and ref. ^23^).

Two hundred and sixty-six studies assessed at least one impact (81%). The most-studied impacts were sales of SSBs/taxed drinks (assessed in 33% of all studies), equity (defined to include variation in any impact by socioeconomic status) and price of SSBs. Sales of SSBs and equity were most often assessed using either observational quantitative or simulation studies, while price of SSBs was almost exclusively assessed through observational quantitative studies.

Twenty-two impacts were assessed in less than 5% of the included studies. A summary of how often each influence and impact was assessed is reported in Supplementary Table 1.

### Studies that considered influences and impacts together

**Fig. 4:**
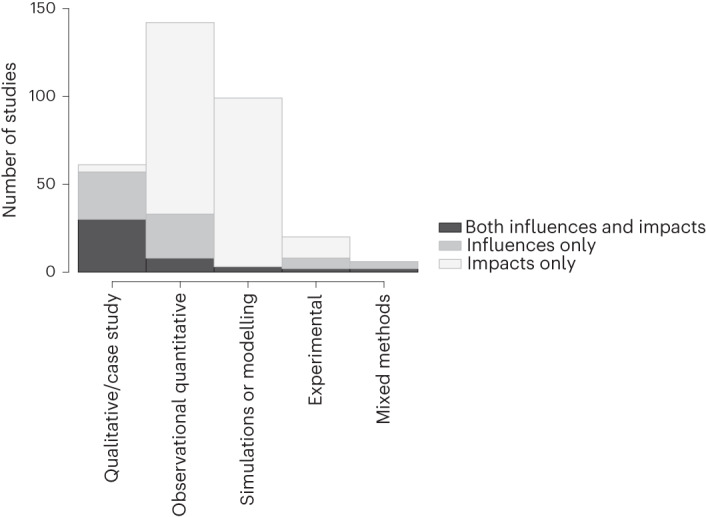
Number of studies assessing impacts only, influences only or both by study type. The figure summarizes the frequency of papers on SSB taxation by study type (qualitative/case study, observational quantitative, simulations or modelling, experimental and mixed methods) and by type of factors assessed (impacts, influences or both).

Forty-five studies (14%) considered influences and impacts of SSB taxation together. The studies that considered both impacts and influences (shown in black in Fig. 4) were primarily qualitative (*n* = 30), together with eight observational quantitative studies, three simulation studies, two experimental studies and two mixed methods studies.

Of the qualitative studies that considered both influences and impacts, Falbe et al.’s analysis of the Berkeley, California, SSB tax provides an illustrative example^24^. The authors conducted semi-structured interviews with key stakeholders following tax introduction and described ways in which the political context influenced implementation of the tax. As the first SSB tax in the United States (influence: political feasibility), there was substantial pressure for it be perceived as a success (impact: perception of effectiveness), leading city officials to invest additional time and resources in the policy—as one city official commented: ‘the whole city is very interested in (making) this is a successful program’^24^. The policy process around tax introduction had direct implications for implementation, both in terms of resources deployed and the urgency of perceived effectiveness.

In another qualitative example, Carriedo et al. used a case study approach to assess the SSB tax in Mexico, informed by policy process theories^25^. They highlighted that in response to tax introduction, industry actors operationalized public–private partnerships, positioning themselves as contributors to national social policy in ways that may ‘jeopardize the policy’ in the future, possibly paving the way for future repeal or policy erosion^25^. In this example, an unintended effect of the tax (impact: industry strategies) may contribute to undermining its sustainability in the future (influence: political feasibility), linking an impact with an influence in a potentially important feedback loop.

Of the quantitative studies that considered both influences and impacts, Buckton et al. provide an illustrative example^26^. They conducted a quantitative content analysis of media coverage around the UK SSB tax and hypothesized that discussion and debate around SSB taxation (impact: awareness) may have contributed to increased public acceptability over time (influence: political feasibility)^26^.

Of the simulation, experimental and mixed methods studies that considered both impacts and influences, all focused on the ways different tax designs (influence: tax design/ease of identifying taxed products) produced varied impacts^27–31^.

### Explicitly systems thinking informed SSB tax evaluations

Of the studies identified, three (<1%) explicitly used systems thinking informed methods. In one study, Urwannachotima et al. (2019) used group model building to identify the dynamic interactions between an SSB tax, sugar consumption and dental caries in Thailand. They found that considering the existing systems around dental health, an SSB tax alone would not be sufficient to achieve the desired level of oral health improvement. The group model-building process enabled stakeholders to identify a range of hypothesized unintended impacts associated with the introduction of an SSB tax, such as potential substitution to other sugary products (an example of policy resistance) and the possible decline in impact of a tax if national incomes increase. The stakeholders suggested that to pre-empt the potential substitution effect from undermining the policy, the ‘tax should be applied to all high sugar content products without exception.’^32^

Building on this group model building work, Urwannachotima et al. (2020) published a second study in which they developed a system dynamics model to estimate the projected impact on dental caries in the population 15 years and older of an SSB tax compared to (1) no intervention and (2) a suite of more comprehensive policy options. They developed three sub-models, focused on caries prevalence, dental service utilization and oral health behaviours, mirroring the causal loop diagram developed in previous work. They found that implementation of the tiered tax on packaged and ready-to-drink SSBs would decrease dental caries 1% by 2040, whereas the suite of policies combined would lead to a 21% decrease over the same time frame. Part of the explanation for the low impact of the SSB tax was that the majority of sugars consumed in the 15+-year-old population are from non-taxed products in Thailand, including from ‘coffee shops and high sugar content desserts and food from street shops,’ and in this context, targeting packaged and ready-to-drink SSBs alone were insufficient^33^.

In the third study, Liu et al. used system dynamics modelling to develop insights concerning the time horizon over which SSB taxation might produce impacts and to consider how tax revenue might be used to maximize childhood obesity prevention efforts^34^. They modelled a perception adjustment delay, noting that consumers may respond to tax-induced price changes gradually. They demonstrated that this delay would result in a greater long-term impact of SSB taxation than may be immediately apparent following implementation. Their models suggest that policymakers should avoid evaluating the success of an SSB tax based on short-term changes in SSB consumption because it may take some time for the full impact of an SSB tax to be realized. Failure to account for this may result in prematurely abandoning a policy that would have otherwise been successful. Liu et al. also considered the impacts of allocating SSB tax revenue to different obesity prevention programmes (for example, subsidizing fruit and vegetables and constructing additional parks to support physical activity), taking into account construction delays and other time-varying factors. They concluded by suggesting that the continued use of system dynamics models in assessing SSB taxation would allow for the identification of implementation dynamics, enable more accurate expectation setting among stakeholders and optimize revenue allocation decisions.

Of the three studies that were explicitly systems informed, all focused on impacts, and none considered both influences and impacts together. Other studies acknowledged the complexity of the systems within which SSB taxes operate^35–37^ but did not explicitly use complexity-informed methods.

### Conceptual synthesis from a systems thinking perspective

**Fig. 5:**
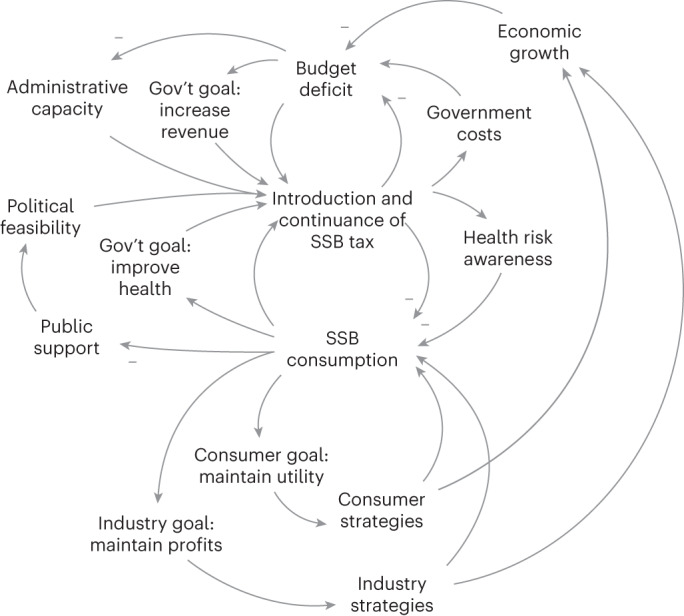
A causal loop diagram based on Sterman’s expanded feedback view of the world. The illustrative framework presented here is not comprehensive but is intended to demonstrate the added value of adopting a feedback-oriented perspective aligned with Sterman’s (2002) ‘feedback view of the world.’ In different settings and at different times, the balance of power between these loops may vary, demonstrating that a variety of behaviours over time are possible. For example, the importance of the health and revenue goals may vary substantially across settings, both in practice and in framing to the public. Depending on which loops dominate, it is possible for the continuance of the tax policy itself to be maintained, strengthened, eroded or repealed over time. Tax repeal would be represented as a complete decrease in the ‘Introduction and continuance of the Tax’ variable. This reflects what we see in practice—SSB taxes have been introduced, increased^59,80^, threatened^81^ and repealed^82^ in a variety of settings. Trends in SSB consumption may also vary depending on which loops prevail, with consumer and industry strategies highlighting potential policy resistance with respect to the health goal. This type of conceptual framework, underpinned by key systems thinking concepts, may enable identification upfront of areas of potential policy resistance. Link polarity is (+) unless otherwise shown.

Building on Sterman’s (2002) expanded ‘feedback view of the world’ (Supplementary Text 1) and the influences and impacts identified through the review, we propose a feedback-oriented conceptual model in Fig. 5.

We have expanded on Sterman’s (2002) model to incorporate key influences and impacts identified in the review. We depict two potential goals for SSB taxation: improving health and reducing the budget deficit (increasing revenue). Accordingly, there are at least two ‘environments’ that an SSB tax may influence: the consumption of SSBs environment (encompassing price of SSBs, number of SSBs, product size and so on) and the fiscal policy environment (encompassing government costs, economic growth, administrative capacity and so on). Making this explicit encourages consideration of which or both goals may be in effect in a given setting. A change in awareness of health risks effect may be an unintended ‘side effect’ of the introduction of an SSB tax, although one that may amplify the impacts from a health perspective, whereas the additional government costs of administering an SSB tax may be an unintended and undesired ‘side effect,’ which could vary based on the tax design.

We also illustrate two groups of actors that may have other goals (for example, companies may aim to maintain or maximize profits through industry strategies while minimizing industry costs; consumers may aim to maintain or maximize utility from SSBs while minimizing household costs/expenditure). As a result, both groups may engage in a variety of strategic responses (for example, through changes in marketing, promotions, sugar concentration, sales/consumption of non-SSBs/untaxed SSBs/low-sugar SSBs, cross-border sales and so on), which themselves go on to shape the SSB environment. This feedback-oriented framework is provisional but aims to illustrate how concepts from the SSB tax literature may map on to a more generic feedback structure, illustrating how what we often think of as ‘influences’ and ‘impacts’ of a policy intervention may be linked through feedback loops.

## Discussion

We set out to consider a wide range of empirical evidence around SSB taxation, through a systems oriented feedback perspective. We identified 329 records for inclusion and 57 hypothesized influences and impacts connected to SSB taxation. Out of the factors considered, a third of all studies assessed the sale of SSBs. Influences were predominantly assessed by qualitative approaches, while a mix of quantitative and simulation approaches were used to assess impacts. Forty-five studies considered influences and impacts together, identifying possibilities of considering both types of factor in an analysis and ‘closing the loop’ on one or more potential feedback loops regarding SSB taxation. We found three studies that explicitly used systems thinking informed methods^32–34^. However, findings from studies that are not explicitly systems thinking informed themselves may still contribute to a systems informed synthesis by informing links within a feedback-oriented framework. We present one such framework in Fig. 5.

### Strengths and limitations

We drew upon a wide range of disciplines (for example, public finance, agricultural economics, health economics, geography, health policy, public health) and used an innovative approach with a focus on feedback loops.

Yet in choosing to focus on peer-reviewed published manuscripts, we may have missed insights from assessments of SSB taxation published in other formats, for example, study protocols^38^, governmental reports on SSB taxes^39^ or civil society reports^40^. We did not extract data on strength of evidence, direction of effect or effect size—a pragmatic decision that was consistent with our aims in this scoping review. Aggregating and disaggregating categories was driven by a thematic ‘clustering’ process: another research team may have arrived at a different number or structure of groupings, with implications for the rankings by frequency. Our findings may underreport the number of systems thinking SSB taxation publications if the studies did not use systems thinking language, methods or references or had a broader scope than SSB taxation*.* For example, some studies were planned as inputs to a broader systems informed evaluation^38^ but did not use systems informed approaches themselves^19,41–44^. These studies were included in the review but not identified as explicitly systems thinking informed. Other systems thinking informed papers only briefly addressed SSB taxation and did not meet our screening criteria, so were excluded^45^. Finally, the last search was run in April 2021, although we do not believe that our findings would be substantially impacted by studies published after this date (Methods, Screening provide more details).

### Policy implications of our proposed conceptual model

There is a growing awareness and interest in the varied ways SSB taxes may arise and produce change within a system. In 2014, Mytton et al. summarized the implicit theoretical framework used in food and beverage tax evaluations, consisting of seven main impacts and no influences^46^. Whereas the authors called for an expansion on this theory of change, the linear, price-driven model continues to be influential^2^. Ng et al. (2021) recently developed a framework for SSB tax evaluations organized by stakeholder groups, highlighting goals for governments and health advocates and potential industry and consumers responses^47^. Several evaluations have also developed systems informed theories of change, such as the SSB tax evaluation in Thailand described above^32,33^ and the UK Soft Drinks Industry Level evaluation^38^. However, we did not identify any evaluations from a systems perspective that considered both impacts and influences of SSB taxation, as recommended in Sterman’s (2002) ‘feedback view of the world.’ We suggest that this presents an important opportunity for a systems thinking informed approach to future evaluations.

Our proposed framework (Fig. 5) could inform the evaluation of a specific SSB tax and the synthesis of multiple types of SSB evidence. Policy evaluations often include multiple work packages that address discrete questions, followed by an effort to ‘bring them together’ in a final work package. We suggest that the initial work package in such an evaluation could focus on theory development using a feedback-oriented framework and generate novel research questions to guide the rest of the evaluation^48^. By linking influences and impacts (which tend to be assessed using different study designs), a feedback model prompts the development of integrative questions, providing a framework for bringing together findings such that ‘the whole is greater than the sum of the parts’ (a key aim of integration). A feedback-oriented approach emphasizes the processes that continue over time that may result in a tax being strengthened, maintained, eroded or repealed. This conceptualization may help to more fully explain and counter potential policy resistance and continue to improve existing SSB tax policies. Our proposed model could be built upon by integrating additional factors, working with setting-specific stakeholder communities and developing a system dynamics model to enable simulation.

## Methods

We used a systematic scoping review method^49,50^ to identify a wide range of literature related to SSB taxation. We describe the literature from a systems informed perspective, with an emphasis on informing a feedback-oriented conceptual framework linking influences, SSB taxation and impacts.

### Search strategy

We used a wide range of electronic databases (MEDLINE via Ovid, Web of Science, Scopus, PsycInfo, Global Health, EconLit, Africa-Wide Information, LILACS, ABI/INFORM Collection, Business Source Ultimate and Google), chosen to maximize the diversity of disciplinary and geographical perspectives.

We tailored search strategies for each database, using terms related to ‘sugar-sweetened beverages’ (for example, soft drinks, soda, fizzy drinks, cola and so on) and taxation (for example, levy, duty, excise, tariff and so on) (Supplementary Text 2). We did not impose any date or language restrictions and conducted all searches on 29 April 2021.

### Screening

Duplicates were removed using Endnote (version 20.0.1, Clarivate), followed by a manual review and finally a process within Covidence (Covidence systematic review software, Veritas Health Innovation; www.covidence.org). Screening was managed in Covidence.

We used a two-stage screening process. In the first stage (title/abstract screening), inclusion criteria were broad: records were included if they mentioned SSB taxation in any way. Records were excluded if they focused on alcohol taxation, sugar taxation (for example, raw sugar, sugar beet and so on), coffee bean/tea leaf/cocoa taxation (for example, no mention of ready-to-drink forms) or value added taxation with no mention of SSBs in particular.

In the second stage (full text screening), we included records reporting original data analysis on SSB taxation published in academic journals and excluded periodicals, blog posts, newspaper articles, review papers and conceptual papers (that is, those without original data analysis).

A sub-sample (5%) of all deduplicated records were reviewed at the title/abstract and full text screening stages by the primary reviewer (M.A.) and secondary reviewers (T.P., L.C.-W.). Following high levels of agreement (kappa score >80%), M.A. completed the remainder of screening.

Searches were re-run over the period 30 April 2021 to 1 May 2023 to give an indication of how the literature may have grown over this period, returning 671 hits (compared to 3,565 studies identified up to 29 April 2021). We searched the title/abstract fields for variations of ‘systems thinking,’ ‘group model,’ ‘causal loop,’ ‘agent based,’ and ‘system dynamics’ to gauge whether there had been an increase in systems informed evaluations of SSB taxes. We identified only one study that was explicitly systems informed and would have met our inclusion criteria: a book chapter summarizing the overall systems informed approach to evaluating the UK Soft Drinks Industry Levy, which described the use of a systems map ‘to hypothesize a wide range of potential impacts of the levy across sectors.’^51^ We also identified a study protocol that described the proposed use of systems thinking in Fiji and Samoa in relation to food policies^52^, an agent-based model of an ultra-processed food tax in Mexico^53^ and a recent review of oral health interventions that called for increased use of systems science^54^. While indicative of the growing interest in systems thinking, these studies would have been excluded on the basis of document type (a study protocol, a review) and the focus on an ultra-processed food tax rather than an SSB tax. Although this is an informal assessment, it provides an indication that systems informed evaluations of SSB taxes remain rare, despite sustained interest^47,54^. We do not anticipate that our conclusions about the value and feasibility of integrating a feedback-oriented perspective into evaluations of PHIs would have substantially changed based on the most recent literature.

### Data extraction

To generate a list of influences and impacts, we initially drew on reviews and conceptual papers identified in the first stage of screening, prioritizing those that considered multiple factors^55–68^. From these, we developed a list of hypothesized influences and impacts using an inductive coding process, followed by clustering to achieve a pragmatic level of abstraction^69^. As described in Supplementary Text 1, we defined ‘influences’ as factors that contributed to the introduction of an SSB tax and ‘impacts’ as factors that resulted from the introduction of a tax.

Then we turned to the included empirical papers identified in the second stage of screening and extracted the following information using Microsoft Excel (Version 2307):

- Authors
- Year published
- Tax setting (for example, national/subnational jurisdiction with the enacted, proposed or hypothetical SSB tax)
- Study type (for example, observational quantitative, qualitative, simulation/modelling, mixed methods, experimental, categorized according to the definitions summarized in Supplementary Table 2)
- Explicitly systems thinking informed (yes/no)
- Hypothesized influences/impacts considered

We defined ‘explicitly systems thinking informed studies’ as those studies that described adopting a systems thinking perspective or demonstrated doing so either by discussing key systems thinking concepts (for example, feedback loops, reference modes), citing key texts^13,70–72^ or applying systems oriented methods and tools, such as social network analysis, agent-based modelling, group model building, causal loop diagrams, stock and flow diagrams, systems archetypes or systems dynamics models^1,73,74^. We note however, that systems informed evaluations do not necessarily need to apply systems methods but may instead pose research questions from a systems perspective and address these using a myriad of appropriate (non-systems) methods^75,76^.

We reviewed each included empirical paper and extracted data on whether the factors identified from the reviews/conceptual papers were assessed (yes/no). We also considered whether any additional factors not previously identified were assessed, which led to the identification of additional influence/impact factors.

We then analysed the full list of factors using ‘clustering,’ a process of abstracting detailed codes^69^, to develop a list of parent factors. Each empirical paper was reviewed a second time against this list of parent factors. All data extraction was initially conducted by M.A. and reviewed by all other authors, with discrepancies resolved through discussion.

### Analysis

We characterized the peer-reviewed evidence using a series of figures describing the overall number of papers identified by date published, study type and factors assessed. We described the subset of papers that considered both influences and impacts and the subset of papers that explicitly used systems methods and describe the types of insight generated in both cases. Finally, we present an example of a feedback-oriented conceptual model to illustrate the potential for combining empirical studies in a systems informed framework.

### Preferred reporting items for systematic reviews and meta-analyses extension for scoping reviews and protocol

Our protocol was prospectively registered on the OpenScience Framework on 13 May 2021 (ref. ^77^) and is reproduced in Supplementary Text 2. We followed the protocol closely, although we did not produce heat maps as intended, given the larger than anticipated range of influences and impacts identified, and developed a stacked bar chart instead. We also modified the stage at which we developed the causal loop diagram (CLD), opting to develop a high-level CLD at the end of the review rather than a preliminary CLD based on every hypothesized link, as described in the protocol. This change reflects our increasing understanding of the utility of CLDs. We followed the preferred reporting items for systematic reviews and meta-analyses extension for scoping reviews checklist (Supplementary Text 3)^78^.

### Reporting summary

Further information on research design is available in the Nature Portfolio Reporting Summary linked to this article.

### Supplementary information

Supplementary Information Supplementary Tables 1 and 2 and Texts 1–3.

Reporting Summary

### Source data

Source Data Fig. 3 Summary of all 329 included papers and data extracted for this review on each paper, including all influences/impacts assessed in each paper.

## Data Availability

The full search strategy in this review has been published in the Supplementary Information. We searched the following databases: MEDLINE via Ovid (https://ospguides.ovid.com/OSPguides/medline.htm), Web of Science (https://clarivate.com/products/scientific-and-academic-research/research-discovery-and-workflow-solutions/webofscience-platform/), Scopus (https://www.elsevier.com/en-in/solutions/scopus), PsycInfo (https://www.apa.org/pubs/databases/psycinfo/), Global Health (https://www.ebsco.com/products/research-databases/global-health), EconLit (https://www.ebsco.com/products/research-databases/econlit), Africa-Wide Information (https://www.ebsco.com/products/research-databases/africa-wide-information), LILACS (https://lilacs.bvsalud.org/en/), Google (www.google.com), ABI/INFORM Collection (https://about.proquest.com/en/products-services/abi_inform_complete/) and Business Source Ultimate (https://www.ebsco.com/products/research-databases/business-source-ultimate). Citations of included studies and extracted data for each paper are available via OSF at 10.17605/OSF.IO/M8F5G. Source data are provided with this paper.

## Code availability

Code used to generate the figures is available upon request.

## References

[1] McGill, E. et al. Evaluation of public health interventions from a complex systems perspective: a research methods review. *Social Sci. Med.*10.1016/j.socscimed.2021.113697 (2021). 10.1016/j.socscimed.2021.113697 33508655

[2] *WHO Manual on Sugar Sweetened Beverage Taxation Policies* (WHO, 2022).

[3] Haque M (2020). A narrative review of the effects of sugar-sweetened beverages on human health: a key global health issue. J. Popul. Ther. Clin. Pharmacol..

[4] *Tackling NCDs: ‘Best Buys’ and Other Recommended Interventions for the Prevention and Control of Noncommunicable Diseases* (WHO, 2017); https://apps.who.int/iris/bitstream/handle/10665/259232/WHO-NMH-NVI-17.9-eng.pdf?sequence=1&isAllowed=y

[5] *World Bank Global SSB Tax Database 2023* (World Bank, accessed 4 April 2023); https://ssbtax.worldbank.org/

[6] Andreyeva T, Marple K, Marinello S, Moore TE, Powell LM (2022). Outcomes following taxation of sugar-sweetened beverages: a systematic review and meta-analysis. JAMA Netw. Open.

[7] Cawley J, Thow AM, Wen K, Frisvold D (2019). The economics of taxes on sugar-sweetened beverages: a review of the effects on prices, sales, cross-border shopping, and consumption. Ann. Rev. Nutr..

[8] Teng, A. M. et al. Impact of sugar-sweetened beverage taxes on purchases and dietary intake: systematic review and meta-analysis. *Obesity Rev.* **24**, 1828–1835 (2019). 10.1111/obr.12868 PMC9285619 31218808

[9] Summan A (2020). The potential global gains in health and revenue from increased taxation of tobacco, alcohol and sugar-sweetened beverages: a modelling analysis. BMJ Glob. Health.

[10] Elliott LM, Dalglish SL, Topp SM (2020). Health taxes on tobacco, alcohol, food and drinks in low- and middle-income countries: a scoping review of policy content, actors, process and context. Int. J. Health Policy Manage..

[11] Carriedo A (2021). The political economy of sugar-sweetened beverage taxation in Latin America: lessons from Mexico, Chile and Colombia. Globalization Health.

[12] Thow AM (2018). Fiscal policy to improve diets and prevent noncommunicable diseases: from recommendations to action. Bull. World Health Org..

[13] Sterman, J. *Business Dynamics: Systems Thinking and Modeling for a Complex World* (Irwin/McGraw-Hill, 2000).

[14] Alvarado M (2019). Assessing the impact of the Barbados sugar-sweetened beverage tax on beverage sales: an observational study. Int. J. Behav. Nutr. Phys. Act..

[15] Friberg, R., Halseth, E. M. S., Frode, S. & Ulsaker, S. A. The effect of cross-border shopping on commodity tax revenue: results from a natural experiment. *Discuss. Paper Ser. Econ.* https://ideas.repec.org/p/hhs/nhheco/2022_009.html (2022).

[16] Alvarado M, Penney TL, Unwin N, Murphy MM, Adams J (2021). Evidence of a health risk ‘signalling effect’ following the introduction of a sugar-sweetened beverage tax. Food Policy.

[17] Campos-Vázquez, R. M. & Medina-Cortina, E. M. Pass-through and competition: the impact of soft drink taxes as seen through Mexican supermarkets. *Latin Am. Econ. Rev.* **28**, 3 (2019).

[18] Etilé, F., Lecocq, S. & Boizot-Szantai, C. Market heterogeneity and the distributional incidence of soft-drink taxes: evidence from France*. European Review of Agricultural Economics.* **48**, 915–939 (2020).

[19] Scarborough P (2020). Impact of the announcement and implementation of the UK soft drinks industry levy on sugar content, price, product size and number of available soft drinks in the UK, 2015–19: a controlled interrupted time series analysis. PLoS Med..

[20] Cornelsen L, Quaife M, Lagarde M, Smith RD (2020). Framing and signalling effects of taxes on sugary drinks: a discrete choice experiment among households in Great Britain. Health Econ..

[21] Meadows, D. Leverage Points: Places to Intervene in a System. *Academy for Systems Change* https://donellameadows.org/archives/leverage-points-places-to-intervene-in-a-system/ (1999).

[22] Sterman, J. D. *System dynamics: systems thinking and modeling for a complex world*. Working Paper Series ESD-WP-2003-01.13-ESD Internal Symposium (Massachusetts Institute of Technology Engineering Systems Division Internal Symposium, 2002).

[23] Alvarado, M. Data Source File 1 SSB Tax papers identified. *OSF.* https://osf.io/j6rsc (2023).

[24] Falbe J (2020). Implementation of the first US sugar-sweetened beverage tax in Berkeley, CA, 2015–2019. Am. J. Public Health.

[25] Carriedo A, Lock K, Hawkins B (2020). Policy process and non-state actors’ influence on the 2014 Mexican soda tax. Health Policy Plan..

[26] Buckton CH (2018). The palatability of sugar-sweetened beverage taxation: a content analysis of newspaper coverage of the UK sugar debate. PLoS ONE.

[27] Segovia J, Orellana M, Sarmiento JP, Carchi D (2020). The effects of taxing sugar-sweetened beverages in Ecuador: an analysis across different income and consumption groups. PLoS ONE.

[28] Lee Y (2020). Health impact and cost-effectiveness of volume, tiered, and absolute sugar content sugar-sweetened beverage tax policies in the United States. Circulation.

[29] Caro JC, Ng SW, Taillie LS, Popkin BM (2017). Designing a tax to discourage unhealthy food and beverage purchases: the case of Chile. Food Policy.

[30] Acton RB, Kirkpatrick SI, Hammond D (2020). How does the probability of purchasing moderately sugary beverages and 100% fruit juice vary across sugar tax structures?. Obesity.

[31] Acton RB, Jones AC, Kirkpatrick SI, Roberto CA, Hammond D (2019). Taxes and front-of-package labels improve the healthiness of beverage and snack purchases: a randomized experimental marketplace. Int. J. Behav. Nutr. Phys. Act..

[32] Urwannachotima N, Hanvoravongchai P, Ansah JP (2019). Sugar-sweetened beverage tax and potential impact on dental caries in Thai adults: an evaluation using the group model building approach. Syst. Res. Behav. Sci..

[33] Urwannachotima N, Hanvoravongchai P, Ansah JP, Prasertsom P, Koh R (2020). Impact of sugar-sweetened beverage tax on dental caries: a simulation analysis. BMC Oral Health.

[34] Liu S, Osgood N, Gao Q, Xue H, Wang Y (2016). Systems simulation model for assessing the sustainability and synergistic impacts of sugar-sweetened beverages tax and revenue recycling on childhood obesity prevention. J. Oper. Res. Soc..

[35] Isett KR, Laugesen MJ, Cloud DH (2015). Learning from New York City: a case study of public health policy practice in the Bloomberg administration. J. Public Health Manage. Pract..

[36] Anaf, J., Fisher, M., Handsley, E., Baum, F. & Friel, S. ‘Sweet talk’: framing the merits of a sugar tax in Australia. *Health Promotion Int.*10.1093/heapro/daaa152 (2021). 10.1093/heapro/daaa152 33496322

[37] Dommarco JAR, de Cosío TG, García-Chávez CG, Colchero MA (2019). The role of public nutrition research organizations in the construction, implementation and evaluation of evidence-based nutrition policy: two national experiences in Mexico. Nutrients.

[38] White, M. *Protocol: Evaluation of the health impacts of the UK Treasury Soft Drinks Industry Levy (SDIL)* (National Institute for Health and Care Research, 2017); https://www.journalslibrary.nihr.ac.uk/programmes/phr/1613001/#/

[39] *Sugar Reduction: Report on Progress Between 2015 and 2019* (Public Health England, 2020); https://www.gov.uk/government/publications/sugar-reduction-report-on-progress-between-2015-and-2019

[40] *The Implementation of Taxation on Sugar Sweetened Beverages by the Government of Barbados: A Civil Society Perspective* (Healthy Caribbean Coalition, 2016); http://www.healthycaribbean.org/wp-content/uploads/2016/07/HCC-SSB-Brief-2016.pdf

[41] Law C (2020). The impact of UK soft drinks industry levy on manufacturers’ domestic turnover. Econ. Human Biol..

[42] Law C (2020). An analysis of the stock market reaction to the announcements of the UK soft drinks industry levy. Econ. Human Biol..

[43] Pell D (2021). Changes in soft drinks purchased by British households associated with the UK soft drinks industry levy: controlled interrupted time series analysis. Brit. Med. J..

[44] Pell D (2019). Support for, and perceived effectiveness of, the UK soft drinks industry levy among UK adults: cross-sectional analysis of the International Food Policy Study. BMJ Open.

[45] Guariguata L (2020). Using group model building to describe the system driving unhealthy eating and identify intervention points: a participatory, stakeholder engagement approach in the Caribbean. Nutrients.

[46] Mytton, O. T., Eyles, H. & Ogilvie, D. Evaluating the Health Impacts of Food and Beverage Taxes. *Curr. Obes. Rep.* **3**, 432–439 (2014). 10.1007/s13679-014-0123-x 26626920

[47] Ng SW, Colchero MA, White M (2021). How should we evaluate sweetened beverage tax policies? A review of worldwide experience. BMC Public Health.

[48] Alvarado M (2022). Making integration foundational in population health intervention research: why we need ‘Work Package Zero’. Public Health.

[49] Arksey H, O’Malley L (2005). Scoping studies: towards a methodological framework. Int. J. Soc. Res. Method..

[50] Levac D, Colquhoun H, O’Brien KK (2010). Scoping studies: advancing the methodology. Implementation Sci..

[51] White, M., Adams, J., Law, C. & Scarborough, P. in *Health Taxes: Policy and Practice* (eds Lauer, J.A., Sassi, F., Soucat, A. & Vigo, A.) 116–125 (World Scientific Publishing Co., 2023).

[52] Webster J (2022). Scaling-up food policies in the Pacific Islands: protocol for policy engagement and mixed methods evaluation of intervention implementation. Nutr. J..

[53] Langellier B (2022). Potential impacts of policies to reduce purchasing of ultra-processed foods in Mexico at different stages of the social transition: an agent-based modelling approach. Public Health Nutr..

[54] Broomhead T, Baker SR (2023). From micro to macro: structural determinants and oral health. Community Dent. Oral Epidemiol..

[55] Popkin BM, Ng SW (2021). Sugar-sweetened beverage taxes: lessons to date and the future of taxation. PLoS Med..

[56] Claudy, M., Doyle, G., Marriott, L., Campbell, N. & O’Malley, G. Are sugar-sweetened beverage taxes effective? Reviewing the evidence through a marketing systems lens. *J. Public Policy Market.*10.1177/0743915620965153 (2020).

[57] Cawley, J., Frisvold, D. & Jones, D. *The Impact of Sugar-Sweetened Beverage Taxes on Purchases: Evidence from Four City-Level Taxes in the U.S*. (NBER, 2019); http://www.nber.org/papers/w26393.pdf 10.1002/hec.4141 33463850

[58] Bahl, R. & Bird, R. *Taxing Sugary Drinks* (International Tax and Investment Center, 2020); https://static1.squarespace.com/static/5a789b2a1f318da5a590af4a/t/5f19f1c05102227e15b6f080/1595535809217/Taxing+Sugary+Drinks.pdf

[59] *Taxes on Sugar-sweetened Beverages: International Evidence* (World Bank, 2020); https://openknowledge.worldbank.org/bitstream/handle/10986/33969/Support-for-Sugary-Drinks-Taxes-Taxes-on-Sugar-Sweetened-Beverages-Summary-of-International-Evidence-and-Experiences.pdf?sequence=6&isAllowed=y

[60] Veliz C, Maslen H, Essman M, Taillie LS, Savulescu J (2019). Sugar, taxes, and choice. Hastings 563 Center Rep..

[61] Lombard M, Koekemoer A (2020). Conceptual framework for the evaluation of sugar tax systems. S. Afr. J. Account. Res..

[62] Cedeno L (2019). Global implementation of soda taxes: is there a better solution for combatting obesity?. Brooklyn J. Int. Law.

[63] Lobstein T, Neveux M, Landon J (2020). Costs, equity and acceptability of three policies to prevent obesity: a narrative review to support policy development. Obesity Sci. Pract..

[64] Bobo J, Wallace TC, Chakraborty S (2019). Are soda taxes good policy for combatting obesity and malnutrition?. Eur. J. Risk Reg..

[65] George A (2019). Not so sweet refrain: sugar-sweetened beverage taxes, industry opposition and harnessing the lessons learned from tobacco control legal challenges. Health Econ. Policy Law.

[66] Grummon AH, Lockwood BB, Taubinsky D, Allcott H (2019). Designing better sugary drink taxes. Science.

[67] Chaloupka FJ, Powell LM, Warner KE (2019). The use of excise taxes to reduce tobacco, alcohol, and sugary beverage consumption. Annu. Rev. Public Health.

[68] Ruhara CM (2021). Strengthening prevention of nutrition-related non-communicable diseases through sugar-sweetened beverages tax in Rwanda: a policy landscape analysis. Glob. Health Action.

[69] Miles, M. B. & Huberman, A. M. *Qualitative Data Analysis: An Expanded Sourcebook* (SAGE Publications, Inc., 1994).

[70] Forrester JW (1997). Industrial Dynamics. J. Oper. Res. Soc..

[71] Hovmand, P. S. in *Community Based System Dynamics* 1–16 (Springer, 2014).

[72] Meadows, D. H. & Wright, D. *Thinking in Systems: A Primer* (ed. Wright, D.) (Chelsea Green Publishing, 2008).

[73] Gates EF, Walton M, Vidueira P, McNall M (2021). Introducing systems- and complexity-informed evaluation. New Directions Eval..

[74] Wilkinson J, Goff M, Rusoja E, Hanson C, Swanson RC (2018). The application of systems thinking concepts, methods, and tools to global health practices: an analysis of case studies. J. Eval. in Clin. Pract..

[75] Rod, N. H. et al. Complexity in epidemiology and public health addressing complex health problems through a mix of epidemiologic methods and data. *Epidemiology*10.1097/EDE.0000000000001612 (2023). 10.1097/EDE.0000000000001612 PMC10712344 37042967

[76] Skivington K (2021). Framework for the development and evaluation of complex interventions: gap analysis, workshop and consultation-informed update. Health Technol. Assess..

[77] Alvarado, M., Adams, J., Penney, T. L. & White, M. Protocol for a systematic mapping review of influences and impacts of sugar-sweetened beverage taxation. *OSF.* https://osf.io/fcxde (2021).

[78] Tricco AC (2018). PRISMA extension for scoping reviews (PRISMA-ScR): checklist and explanation. Ann. Intern. Med..

[79] Page MJ (2021). The PRISMA 2020 statement: an updated guideline for reporting systematic reviews. BMJ.

[80] *Barbados Increases Tax on Sugar-Sweetened Beverages* (Global Health Advocacy Incubator, 2022); https://advocacyincubator.org/2022/05/02/barbados-increases-tax-on-sugar-sweetened-beverage/

[81] Narayan, Jyoti. UK PM Truss preparing to scrap sugar tax on soft drinks. *Reuters* https://www.reuters.com/world/uk/uk-pm-truss-preparing-scrap-sugar-tax-soft-drinks-times-2022-09-15/ (15 September 2022).

[82] Powell LM, Leider J (2020). Evaluation of changes in beverage prices and volume sold following the implementation and repeal of a sweetened beverage tax in Cook County, Illinois. JAMA Netw. Open.

