# Supplementary material for: A systematic scoping review evaluating sugar-sweetened beverage taxation from a systems perspective
Source: Nat Food. 2023 Oct 19;4(11):986–95. doi: 10.1038/s43016-023-00856-0 (PMC10661741; doi:10.1038/s43016-023-00856-0)
Supplement: Supplementary file 1 — Supplementary Tables 1 and 2 and Texts 1–3. [file 43016_2023_856_MOESM1_ESM.pdf]

nature food

Article

https://doi.org/10.1038/s43016-023-00856-0

A systematic scoping review evaluating sugar-sweetened beverage taxation from a systems perspective

In the format provided by the authors and unedited

Supplementary Information

Contents

Supplementary Information 1

Supplementary Information Table 1: Definitions of Impacts/Influences considered 2

Supplementary Information Table 2: Definitions for Study Type 5

Supplementary Information: Text 1 6

Supplementary Information Text 2: OSF Protocol 8

Introduction 9

Challenges w/ evaluating population health interventions 9

SSB Taxation as a Case Study 9

Overall aims 9

Methods 9

Eligibility criteria 9

Information sources 10

Search strategy 10

Study records 17

Data extraction Stage 1: Assigning records to 'bins' 17

Data extraction Stage 2: Developing complexity-informed theory 18

Data extraction Stage 3: Mapping the evidence to the complexity-informed theory 19

Supplementary Information Text 3: Preferred Reporting Items for Systematic reviews and Meta-Analyses extension for Scoping Reviews (PRISMA-ScR) Checklist 20

References 24

Supplementary Information Table 1: Definitions of Impacts/Influences considered

	Factors	Definition	Year	Assessed in records	
				x%	#s
Influences	public support	When the focus is on determinants or levels of public support/opposition towards an SSB tax.	2012	16.5%	54
	industry strategies influence	When the SSB industry (e.g. manufacturers, grocers, etc) engage in strategies to influence the introduction of an SSB tax (e.g. before a tax is introduced)	2011	14.6%	48
	political feasibility influence	Broadly refers to the policy process surrounding the introduction of an SSB tax not otherwise specified.	2011	14.3%	47
	tax design/ease of identifying taxed products	When the focus is on how the policy process led to decisions around what kind of tax design (e.g. ad valorem/specific, rate, definition of taxable products, etc.) Does not include simulation studies which consider multiple tax designs, but rather policy process studies, comparative case studies, etc.	2011	10.7%	35
	health	When health (e.g. high obesity rates, high type II diabetes burden, etc.) is identified as part of the motivation for the introduction of an SSB tax (not public perception of a health as the reason for an SSB tax)	2011	8.2%	27
	budget deficit	When a budget deficit is identified as part of the motivation for the introduction of an SSB tax (not, public perception of a budget deficit as the reason for an SSB tax)	2011	6.1%	20
	administrative capacity	When the administrative capacity impacts the introduction or design of an SSB tax.	n/a	0.0%	0
	baseline SSB consumption	When baseline levels of SSB consumption impacts the introduction or design of an SSB tax.	n/a	0.0%	0
	monopolistic SSB market	When the structure of the SSB market (monopolistic, competitive, oligopolistic, etc.) impacts the introduction or design of an SSB tax.	n/a	0.0%	0
Impacts	sales of SSBs/taxed drinks	Changes in sales only of SSBs/taxed products (note, consumption and calories of SSBs are captured elsewhere)	2005	33.2%	109
	equity	Outcomes around any other impact stratified by SES. Does not include other form of equity (e.g. does not include stratifications by male/female or age)	2008	22.9%	75
	price of SSBs	Changes in the price of SSBs	2011	17.7%	58
	sales/consumption of non-SSBs	Changes in sales/consumption of non-SSBs (excluding milk and water, which are captured separately)	2010	16.5%	54
	consumption of SSB	Outcomes around changes in consumption of SSBs - note that many studies conflate purchase of SSBs and consumption of SSBs - aim to use this outcome only for studies which use consumption-specific data such as food frequency questionnaires or 24-hour recalls. Studies which use scanner data, household expenditure data, etc. can be cateogirzed under 'SSB sales'	2010	13.7%	45
	sales/consumption of water	Outcomes around changes in water	2011	13.7%	45
	BMI/obesity rate/bodyweight	Any outcome around BMI/obesity/bodyweight changes after the introduction of an SSB tax.	2008	13.7%	45
	calories/added sugars from SSBs	Outcomes measuring changes in calories or added sugars from SSBs - distinct from total calories. Note	2010	13.1%	43

		that some papers assume changes in SSB-derived categories equate to changes in total calories of the same magnitude, without taking into account potential substitution to non-SSB or food-derived calories. Aim to preserve 'total calories' for studies which take into account full range of substitutions, and use this outcome for studies which only focus on beverage-derived changes.			
	NCDs and cost savings	Any outcome around health (e.g. dental caries, diabetes, QALYs, DALYs, life years gained, etc), excluding BMI, after the introduction of an SSB tax.	2012	11.9%	39
	revenue	Changes in government revenue	2010	11.6%	38
	price of non-SSBs/untaxed beverages	Changes in the price of non-SSBs and/or untaxed beverages	2011	11.6%	38
	sales/consumption/calories from untaxed SSBs	Changes in any outcome (sales, consumption, calories) from untaxed SSBs, such as powdered sweetened drinks in some settings, or sweetened flavoured milks, etc (definition of untaxed SSBs vary from setting to setting)	2012	9.8%	32
	sales/consumption of milk	Changes in sales/consumption of milk	2010	9.8%	32
	gov't costs	Administrative costs of operating an SSB tax, as well as government costs to cover healthcare related expenses.	2012	8.5%	28
	awareness	Outcomes around changes in perceptions of SSBs brought on because of the introduction of an SSB tax. Includes awareness that SSBs are taxed, as well as awareness that SSBs are a health risk (e.g. risk signalling)	2013	8.5%	28
	industry strategies	Industry strategies after an SSB tax has been introduced (excluding marketing, promotions, etc which are separate headings). May include industry efforts to repeal a tax	2008	8.2%	27
	hh costs/expenditure	Household expenditure on SSBs (does not include household expenditure on future healthcare costs)	2010	7.6%	25
	total caloric consumption	Changes in total caloric consumption (note this should be applied to studies which account for substitution to other foods)	2010	7.0%	23
	sales/consumption of low-sugar SSBs	Outcomes around changes in low-sugar SSBs (e.g. especially relevant in evaluations of tiered SSB tax designs)	2018	6.4%	21
	heterogeneity of price change	Outcomes which explicitly address heterogeneous price changes following the introduction of an SSB tax	2015	5.5%	18
	sugar concentration	Changes in sugar concentration of drinks - e.g. reformulation	2017	4.9%	16
	perception of tax effectiveness	Public or stakeholder perception of SSB tax effectiveness after an SSB tax has been introduced (e.g. not effectiveness of SSB taxes in general, but of a specific tax in a specific setting)	2017	4.3%	14
	cross-border sales	Outcomes around changes in SSB sales in neighbouring jurisdictions (e.g. when the neighbouring jurisdiction is not used as a control but is a subject of study on its own)	2017	4.0%	13
	sales of other foods	Changes in sales/consumption of foods	2010	3.7%	12
	diet quality	Outcomes around changes in diet beyond changes in added/total sugar or total calories - may include changes in fat/sodium/carbohydrates/ vitamins, or may also refer to a more holistic 'diet quality' variable	2010	2.7%	9

	perception of price increase	The public's perception of a price change (whether or not it is attributed to the SSB tax)	2017	2.4%	8
	sales of alcohol	Changes in sales of alcohol	2013	2.4%	8
	industry costs	Industry costs in response to the introduction of an SSB tax	2010	2.1%	7
	small business profit	Changes in small business profitability	2017	1.8%	6
	other taxes/policies	Changes in the political feasibility of other taxes or policies	2014	1.8%	6
	political feasibility	Changes in the political feasibility of increasing/maintaining/changing an SSB tax after it has been introduced.	2018	1.8%	6
	economic growth	Outcomes around productivity, GDP, etc. (excludes changes in employment)	2017	1.8%	6
	total consumption of added sugars	Outcomes around changes in added sugars overall (e.g. not just from SSBs)	2016	1.8%	6
	number of SSBs	Changes in the number of SSB products on the market (e.g. introduction of new products, consolidation of existing ones, etc.)	2018	1.5%	5
	jobs	Changes in jobs/employment following the introduction of an SSB tax	2014	1.5%	5
	utility from SSBs	Changes in utility/benefit derived from drinking SSBs	2012	1.5%	5
	number of non-SSBs	Changes in the number of non-SSB products on the market (e.g. introduction of new products, consolidation of existing ones, etc.)	2013	1.5%	5
	product size	Changes in the product sizes available	2008	1.5%	5
	promotions	Changes in promotions (including gifts, bundling, discounted prices)	2020	1.2%	4
	marketing	Changes in marketing (broadly defined) following the introduction of an SSB tax	2020	0.9%	3
	SSB imports	Any outcome around changes to SSB imports after an SSB tax	2020	0.6%	2
	sugary food imports	Any outcome around changes to imports of other sugary products after an SSB tax	2021	0.3%	1
	autonomy	When consumer autonomy has been assessed following the introduction of an SSB tax	n/a	0.0%	0
	NNS advertising	Any outcome around the level/type of advertising around NNS (or non-SSBs) after an SSB tax	n/a	0.0%	0
	legal action threats	Any outcome of the level or existence of legal action after an SSB tax	n/a	0.0%	0
	resources for evaluation	Any outcome on the allocation of resources for evaluation of SSB taxes	n/a	0.0%	0
	use of prime shelf space for SSBs	Any outcome on the use of prime shelf space for SSBs after an SSB tax	n/a	0.0%	0
	point-of-purchase tax labels	Any outcome on presence or level of point-of-purchase tax labels	n/a	0.0%	0

Supplementary Information Table 2: Definitions for Study Type

	Category	Definition
Study Type	Experimental	Studies involving manipulation of conditions by the research team such as virtual supermarket trials or vending machine experiments
	Qualitative or case study	Studies involving qualitative approaches, such as interviews, stakeholder analysis, documentary analysis and case studies
	Observational quantitative	Studies involving quantitative approaches, including interrupted time series analysis, difference in difference analysis, regression analysis, etc.
	Simulations or modelling	Studies involving quantitative modelling approaches, including microsimulation, demand models, systems dynamics models
	Mixed methods	Studies involving both quantitative and qualitative methods.

Supplementary Information: Text 1

In the context of population health intervention (PHI) evaluations, it may be helpful initially to differentiate between two main types of factors, which we will call 'influences' and 'impacts.' We use this terminology to distinguish between factors that are precursors to the PHI and contribute to causing the introduction or continued existence of it ("influences") and factors which are caused by the PHI, either as short- or long-term consequences ("impacts"). See Sterman (2002) Exhibit 3 for a generic example of a linear representation of the links between a goal, a situation, a problem, a decision and results.[1]

Sterman reimagines this theory of change through a single feedback loop (see the top half of Exhibit 4 in Sterman 2002), suggesting that "the results of our actions define the situation we face in the future. The new situation alters our assessment of the problem and the decisions we take tomorrow."[1] In this simple conception, changes in outcomes are embedded within changes to the environment, which influence future decisions (e.g. maintain, increase, scrap the policy). This type of simple feedback loop is exemplified by a model which demonstrated that if tobacco control measures reduce the number of smokers, this reduction will lead to greater support for even more tobacco control measures, creating a simple but potentially powerful re-enforcing feedback loop between impacts and influences of a PHI intervention.[2]

Sterman builds on the simple single-loop model in the lower half of Exhibit 4, highlighting that our decisions also lead to unintended side effects, while changes in the environment feed back into the original goals, the goals of other agents, and the actions of others in ways which may, themselves, reshape the environment.[1] As Sterman summarizes:

> *"…our actions may also trigger side effects we didn't anticipate. Other agents, seeking to achieve their goals, react to restore the balance we have upset. Policy resistance arises because we do not understand the full range of feedbacks operating in the system."*[1]

Often, we do not account for feedback loops when designing and evaluating potential PHIs, especially when feedback occurs with a delay or involves elements of the systems beyond the intended theory of change. Doing so may help us to identify and curtail potential sources of policy resistance.

The distinction between influences and impacts becomes blurred when viewed through a feedback lens– both types of factors are likely connected such that the impact of a PHI may eventually have an effect on an influencing factor and so on. Ross's detailed account of UK drunk driving policy provides an illustrative example.[3] After the UK's Road Traffic Act of 1962 was perceived to have been ineffective at reducing drunk driving casualties, support grew to amend the policy, influencing the development of the Road Safety Act of 1967 which introduced a fixed blood alcohol limit and compulsory testing. The introduction of the 1967 law was well publicized and led to the intended impact – decreased casualties, at least at first. However, police enforcement was low (because of the complex processes in gaining a conviction and low perceived importance of drunk driving infractions). Over time, people realized that the threat of punishment was minimal and resumed their previous behaviours, ultimately causing casualty rates to increase to their original levels. The initial policy effectiveness and subsequent rebound informed future debate about the UK's subsequent drunk driving policy, leading to the recommendations to address police enforcement and introduce random checks, further feeding back into the policy process.[3] In this richly described example, the links between the impacts (or lack of

impacts) of a policy clearly become influences in the next policy iteration. There are likely parallels between this example and many other PHIs.

However, contemporary PHI evaluation literature is broadly divided into studies that consider influences (causally relevant factors which precede the PHI) or impacts (causal consequences which follow on from the PHI), most often using quite different methods. Therefore, to build an understanding of the feedbacks between the two, it is useful to first develop a nuanced understanding of the influences and impacts concerning a PHI. We suggest that considering influences and impacts together may then help to 'close the loops,' by connecting factors which had previously been conceptualized linearly.

There are many other aspects of systems thinking, such as accumulations over time, delays, emergence, adaptation, and considering an intervention as a disruption to a pre-existing or underlying system. Here, we focus specifically on feedback loops as a key dimension of systems thinking, acknowledging that this is only a starting point. However, this approach may contribute meaningfully to the growing body of evidence around various PHIs by encouraging researchers to ask a different set of research questions with the aim of understanding and potentially countering potential policy resistance.[4] Causal loop diagrams (CLDs) are one systems thinking tool that can help to map and visualize feedback loops between components of a system.[5]

Supplementary Information Text 2: OSF Protocol

Protocol for a systematic mapping review of influences and impacts of sugar-sweetened beverage taxation

Date: May 13, 2021

Authors:

- Miriam Alvarado (corresponding author),[1,3] mra47@cam.ac.uk, +44 7944524573
- Jean Adams,[1] jma79@medschl.cam.ac.uk
- Tarra L Penney,[2] tpenney@yorku.ca
- Martin White,[1] martin.white@mrc-epid.cam.ac.uk

[1]Centre for Diet and Activity Research, MRC Epidemiology Unit, University of Cambridge School of Clinical Medicine, Box 285 Institute of Metabolic Science, Cambridge Biomedical Campus, Cambridge, CB2 0QQ, United Kingdom

[2]Global Health Program, Faculty of Health, York University, 4700 Keele Street, Toronto, Canada

[3]George Alleyne Chronic Disease Research Centre, Caribbean Institute for Health Research, The University of the West Indies, Bridgetown, Barbados

Roles and responsibilities:

- Content expertise: MA, JA, TP, MW all have SSB taxation content expertise
- Systematic review methods: MA, JA, TP, MW
- Statistical analysis: MA, JA, TP, MW
- Information retrieval: Isla Kuhn was consulted as an information specialist (ilk21@cam.ac.uk, University of Cambridge)

Introduction

Challenges w/ evaluating population health interventions

Population health interventions hold the promise of being able to effect change on a large scale. They are also often complex interventions operating within complex systems. The methods we often use to synthesize evidence on interventions may not be fit-for-purpose, if we are interested in understanding how these complex interventions work and how they could work better.

SSB Taxation as a Case Study

Here, we propose and pilot an approach to developing a complexity-informed understanding of how one intervention 'works.' We then use this complexity-informed theory to critically assess the empirical literature and identify gaps in the evidence base. We use SSB taxation as an exemplar complex intervention.

SSB taxes are a complex intervention because 1) they target a high number of products (e.g. heterogenous types of SSBs) and organizations (SSB manufacturers, importers and distributors); 2) there are multiple and variable outcomes of interest (e.g. SSB consumption amongst different groups – SES, high/low consumers, age, etc., total caloric consumption, environmental impacts), and 3) SSB taxes themselves vary quite a bit across settings (e.g. different tax designs, definitions of taxable products, etc.).[6] The policy processes by which SSB tax are introduced may impact both the mechanisms of effect and the design of the tax.

A complexity-informed understanding of SSB taxes as an intervention would allow us to more efficiently direct research resources to addressing unanswered questions, allow us to anticipate and/or monitor unintended consequences, and to explain heterogeneity between taxes and outcomes more completely.

Overall aims

Our aims are to:

- Develop complexity-informed theory around SSB taxation
- Identify gaps in the empirical literature

We aim to identify the ways in which SSB taxes impact and are impacted by the underlying systems within which they are introduced and operate, considering differences in tax structure and context. We are interested in both theoretical and empirical impacts. We are also interested in both determinants of SSB taxation (e.g. the policy process) and the impacts of an SSB tax (e.g. impact evaluations).

We intend to conduct a systematic conceptual[7] and evidence mapping review[8,9] to assess the extent to which the empirical evidence base aligns with complexity-informed theory and identify gaps in the evaluation literature.

Methods

Eligibility criteria

Sources will be included if they evaluated, simulated or theorized about factors which either led to the introduction of an SSB tax, or resulted from an SSB tax.

Inclusion criteria:

- Includes a focus on sugar-sweetened beverage taxes
- Focus can be broad, for example:
    - About sugar-sweetened beverages and related taxes
    - About indirect taxation (e.g. excise, import, sales) and mentions SSBs

Exclusion Criteria

- Exclusively about alcohol taxation (e.g. beer, wine, etc.)
- Exclusively about sugar taxation (e.g. raw sugar, sugarcane, sugar beet, etc.)
- Exclusively about coffee beans/tea leaves/cocoa taxation – no mention of ready-to-drink form and taxation
- Exclusively about value-added taxation (not specific to SSBs)
- Exclusively about food taxation
- Exclusively about corporate taxation, income taxation
- Exclusively about fuel/gasoline taxation
- Exclusively about drinking water, with no discussion of indirect taxation
- Newspaper, blog, periodical document types

We will not impose any date or language restrictions.

Information sources

We will use the electronic databases summarized in Table 1.

Table 1: Databases

**Database**	**Rationale for inclusion**
MEDLINE via Ovid	For health perspective
Web of Science	For broad perspective
Scopus	For broad perspective
PsycInfo,	For psychology perspective
Global Health	To increase global coverage
EconLit	For economics perspective
Africa-wide Information	To increase global coverage
LILACS	To increase global coverage
Google	To identify grey literature
ABI/INFORM Collection	For business perspectives
Business Source Ultimate	For business perspectives

Search strategy

We will tailor search strategies for each database, broadly searching for terms related to “sugar sweetened beverages” (e.g. soft drinks, soda, fizzy drinks, cola, etc.) and taxation (e.g. levy, duty, excise, tariff, etc.)

We will also consult with experts to identify additional records.

Sample Indicator papers

- Sugar-sweetened beverage taxes: Lessons to date and the future of taxation
- Changes in soft drinks purchased by British households associated with the UK soft drinks industry levy: controlled interrupted time series analysis
- Distribution of sugar-sweetened beverage sales volume by sugar content in the United States: implications for tiered taxation and tax revenue
- The political economy of sugar-sweetened beverage taxation in Latin America: lessons from Mexico, Chile and Colombia
- The Impact of Tax on Non-Alcoholic Beverage Demand.
- https://openknowledge.worldbank.org/bitstream/handle/10986/28569/120225-WP-P154568-ColombiaPoliticalEconomy-PUBLIC.pdf?sequence=1
- https://www.paho.org/en/node/78468
- https://www.jhsph.edu/departments/health-behavior-and-society/_pdf/Advocating_For_Sugar_Sweetened_Beverage_Taxation.pdf

Medline via Ovid

Dates: April 29, 2021
Years: Any
Language: Any
Ovid MEDLINE(R) and Epub Ahead of Print, In-Process, In-Data-Review & Other Non-Indexed Citations, Daily and Versions(R) <1946 to April 28, 2021>
1 taxes/ 6873
2 (tariff* or tax or taxes or taxation or excise or excises or duty or duties or levy or levies).mp. 62275
3 1 or 2 62275
4 beverages/ or Artificially Sweetened Beverages/ or exp carbonated beverages/ or coffee/ or exp drinking water/ or energy drinks/ or "fruit and vegetable juices"/ or milk/ or Sugar-Sweetened Beverages/ or tea/ or kombucha tea/ or teas, herbal/ 102366
5 (drink* or beverage* or fizzy or sugar* or sweetened or soda or cola or coke or carbonated or non-alcohol* or nonalcohol*).mp. 409118
6 4 or 5 477047
7 alcohol*.mp. 440644
8 (fizzy or sugar* or sweetened or soda or cola or coke or carbonated or non-alcohol* or nonalcohol* or soft).mp. 423043
9 7 and 8 40984
10 7 not 9 399660
11 (3 and 6) not 10 1145

Web of Science

Dates: 29 April 2021
Years: Any
Language: Any
TS=(“tax” OR “taxes” OR “taxation” OR “taxing” OR “taxed” OR “tariff” OR “tariffs” OR “duty” OR “duties” OR “excise” OR “excises” OR “levy” OR “levies”)

TS =(“drink” OR “drinks” OR “beverage” OR “beverages” OR “fizzy” OR “sugar” OR “sugary” Or “sugars” OR “sweetened” OR “soda” OR “cola” OR “coke” OR “carbonated”)
3 TS=alcohol*
4 TS= (“fizzy” OR “sugar” OR “sugary” Or “sugars” OR “sweetened” OR “soda” OR “cola” OR “coke” OR “carbonated” OR non-alcohol* OR nonalcohol* OR “soft”)
5 #3 AND #4
6 #3 NOT #5
7 (#1 AND #2) NOT #6
AND DOCUMENT TYPES: (Article OR Book OR Book Chapter OR Data Paper OR Database Review OR Meeting Abstract OR Meeting Summary OR Proceedings Paper OR Review)

Scopus

Dates: 29 April 2021
Years: Any
Language: Any
1 TITLE-ABS-KEY ({tax} OR {taxes} OR {taxation} OR tariff* OR {duty} OR {duties} OR {excise} OR {excises} OR {levy} or {levies})
2 TITLE-ABS-KEY ({drink} OR {drinks} OR beverage* OR {fizzy} OR {sugar} OR {sugary} OR {sugars} OR {sweetened} OR {soda} OR {cola} OR {coke} OR {carbonated})
3 TITLE-ABS-KEY (alcohol*)
4 TITLE-ABS-KEY ({fizzy} OR {sugar} OR {sugary} OR {sugars} OR {sweetened} OR {soda} OR {cola} OR {coke} OR {carbonated} OR non-alcohol* OR nonalcohol* OR {soft})
5 #3 AND #4
6 #3 AND NOT #5
7 (#1 AND #2) AND NOT #6

AND (LIMIT-TO (DOCTYPE , "ar") OR LIMIT-TO (DOCTYPE , "re") OR LIMIT-TO (DOCTYPE , "cp") OR LIMIT-TO (DOCTYPE , "ch") OR LIMIT-TO (DOCTYPE , "bk") OR LIMIT-TO (DOCTYPE , "cr") OR LIMIT-TO (DOCTYPE , "ab"))

PsycINFO

Dates: 29 April 2021
Years: Any
Language: Any
1 DE “taxation”
2 TI (tariff* or tax or taxes or taxation or excise or excises or duty or duties or levy or levies) OR AB (tariff* or tax or taxes or taxation or excise or excises or duty or duties or levy or levies) OR KW (tariff* or tax or taxes or taxation or excise or excises or duty or duties or levy or levies)
3 S1 or S2
4 DE "Beverages (Nonalcoholic)" OR DE "Energy Drink" OR DE "Drinking Behavior"
5 TI (drink* or beverage* or fizzy or sugar or sugars or sugary or sweetened or soda or cola or coke or carbonated) OR AB (drink* or beverage* or fizzy or sugar or sugars or sugary or sweetened or soda or cola or coke or carbonated) OR KW (drink* or beverage* or fizzy or sugar or sugars or sugary or sweetened or soda or cola or coke or carbonated)
6 S4 or S5
7 TI alcohol* OR AB alcohol* OR KW alcohol*

8 TI (fizzy or sugar* or sweetened or soda or cola or coke or carbonated or non-alcohol* or nonalcohol* or soft) OR AB (fizzy or sugar* or sweetened or soda or cola or coke or carbonated or non-alcohol* or nonalcohol* or soft) OR KW (fizzy or sugar* or sweetened or soda or cola or coke or carbonated or non-alcohol* or nonalcohol* or soft)

9 S7 and S8

10 S7 not S9

11 (S3 and S6) not S10

Global Health

Dates: 29 April 2021

Years: Any

Language: Any

1 (DE "indirect taxation" OR DE "levies" OR DE "sales tax" OR DE "stamp duty" OR DE "tariffs" OR DE "taxes" OR DE "value added tax")

2 TI (tariff* or tax or taxes or taxation or excise or excises or duty or duties or levy or levies) OR AB (tariff* or tax or taxes or taxation or excise or excises or duty or duties or levy or levies)

3 S1 or S2

4 DE "beverages" OR DE "fruit drinks" OR DE "lactic beverages" OR DE "soft drinks" OR DE "cocoa beverages" OR DE "coconut milk" OR DE "coffee" OR DE "drinking water" OR DE "fruit juices" OR DE "herbal teas" OR DE "mate" OR DE "milk shakes" OR DE "tea" OR DE "vegetable juices"

5 TI (drink* or beverage* or fizzy or sugar or sugars or sugary or sweetened or soda or cola or coke or carbonated) OR AB (drink* or beverage* or fizzy or sugar or sugars or sugary or sweetened or soda or cola or coke or carbonated)

6 S4 or S5

7 TI alcohol* OR AB alcohol*

8 TI (fizzy or sugar* or sweetened or soda or cola or coke or carbonated or non-alcohol* or nonalcohol* or soft) OR AB (fizzy or sugar* or sweetened or soda or cola or coke or carbonated or non-alcohol* or nonalcohol* or soft)

9 S7 and S8

10 S7 not S9

11 (S3 and S6) not S10

Source type:

- Academic Journals
- Books
- Reports
- Conference Proceedings

EconLit

1 TI (“tax” OR “taxes” OR “taxation” OR tariff* OR “duty” OR “duties” OR “excise” OR “excises” OR “levy” or “levies”) OR AB (“tax” OR “taxes” OR “taxation” OR tariff* OR “duty” OR “duties” OR “excise” OR “excises” OR “levy” or “levies”)

2 TI (“drink” OR “drinks” OR beverage* OR “fizzy” OR “sugar” OR “sugary” OR “sugars” OR “sweetened” OR “soda” OR “cola” OR “coke” OR “carbonated”) OR AB (“drink” OR “drinks” OR

beverage* OR “fizzy” OR “sugar” OR “sugary” OR “sugars” OR “sweetened” OR “soda” OR “cola” OR “coke” OR “carbonated”)
3 TI alcohol* OR AB alcohol*
4 TI (“fizzy” OR sugar* OR “sweetened” OR “soda” OR “cola” OR “coke” OR “carbonated” OR non-alcohol* OR nonalcohol* OR “soft”) OR AB(“fizzy” OR sugar* OR “sweetened” OR “soda” OR “cola” OR “coke” OR “carbonated” OR non-alcohol* OR nonalcohol* OR “soft”)
5 S3 AND S4
6 S3 NOT S5
7 (S1 AND S2) NOT S6

Africa-wide Info

1 TI (tax OR taxes OR taxation OR tariff* OR duty OR duties OR excise OR excises OR levy or levies) OR AB (tax OR taxes OR taxation OR tariff* OR duty OR duties OR excise OR excises OR levy or levies)
2 TI (drink OR drinks OR beverage* OR fizzy OR sugar OR sugary OR sugars OR sweetened OR soda OR cola OR coke OR carbonated) OR AB (drink OR drinks OR beverage* OR fizzy OR sugar OR sugary OR sugars OR sweetened OR soda OR cola OR coke OR carbonated)
3 TI alcohol* OR AB alcohol*
4 TI (fizzy OR sugar* OR sweetened OR soda OR cola OR coke OR carbonated OR non-alcohol* OR nonalcohol* OR soft) OR AB (fizzy OR sugar* OR sweetened OR soda OR cola OR coke OR carbonated OR non-alcohol* OR nonalcohol* OR soft)
5 S3 AND S4
6 S3 NOT S5
7 (S1 AND S2) NOT S6

Source Types

- Academic Journals
- Books
- Reports
- Conference Papers

LILACS

Dates: 30 April 2021
Years: Any
Language: Any
Title, abstract, subject -
1 (tax OR taxes OR taxation OR tariff* OR duty OR duties OR excise OR excises OR levy OR levies)
2 (drink OR drinks OR beverage* OR fizzy OR sugar* OR sweetened OR soda OR cola OR coke OR carbonated)
3 (alcohol*)
4 (fizzy OR sugar* OR sweetened OR soda OR cola OR coke OR carbonated OR non-alcohol* OR nonalcohol* OR soft)
5 (S3 AND S4)
6 (S3 AND NOT S5)

7 (S1 AND S2) AND NOT S6

Business Source Ultimate

Dates: 22 April 2021 -
Maybe update search strategy in Business Ultimate to include “” to prevent carbon, etc.
Years: Any
Language: Any
Limiters - Publication Type: Academic Journal, Book, Case Study, Conference Paper, Conference Proceeding, Dissertation, Grey Literature, Report, Review, Working Paper;

1 DE ("INDIRECT taxation" OR "TARIFF" OR "TAXATION of articles of consumption" OR "EXCISE tax" OR "SALES tax" OR "CONSUMPTION tax")

2 TI (“tax” OR “taxes” OR “taxation” OR tariff* OR “duty” OR “duties” OR “excise” OR “excises” OR “levy” OR “levies”) OR AB(“tax” OR “taxes” OR “taxation” OR tariff* OR “duty” OR “duties” OR “excise” OR “excises” OR “levy” OR “levies”) OR KW(“tax” OR “taxes” OR “taxation” OR tariff* OR “duty” OR “duties” OR “excise” OR “excises” OR “levy” OR “levies”)

3 S1 OR S2

4 DE ("BOTTLED water industry" OR "CARBONATED beverage industry" OR "FRUIT drink industry" OR "FRUIT juice industry" OR "MINERAL water industry" OR "NON-alcoholic beverage industry" OR "SOFT drink industry" OR "VEGETABLE drink industry" OR "VEGETABLE juice industry" OR "DRINK concentrates" OR "BEVERAGE industry distributors" OR "BEVERAGE processing plant costs" OR "BEVERAGE industry statistics" OR "FRUIT juice industry statistics" OR "BOTTLED water industry" OR "CARBONATED beverage industry" OR "BEVERAGE industry" OR "BEVERAGE consumption statistics" OR "SUPERMARKETS -- Beverage departments" OR "FUNCTIONAL beverage sales & prices" OR "BEVERAGE consumption")

5 TI(“drink” OR “drinks” OR beverage* OR “fizzy” OR “sugar” OR “sugary” OR “sugars” OR “sweetened” OR “soda” OR “cola” OR “coke” OR “carbonated”) OR AB(“drink” OR “drinks” OR beverage* OR “fizzy” OR “sugar” OR “sugary” OR “sugars” OR “sweetened” OR “soda” OR “cola” OR “coke” OR “carbonated”) OR KW(“drink” OR “drinks” OR beverage* OR “fizzy” OR “sugar” OR “sugary” OR “sugars” OR “sweetened” OR “soda” OR “cola” OR “coke” OR “carbonated”)

6 S4 OR S5

7 TI(alcohol*) OR AB (alcohol*) OR KW(alcohol*)

8 TI(“fizzy” OR “sugar” OR “sugary” OR “sugars” OR “sweetened” OR “soda” OR “cola” OR “coke” OR “carbonated” OR non-alcohol* OR nonalcohol* OR “soft”) OR AB(“fizzy” OR “sugar” OR “sugary” OR “sugars” OR “sweetened” OR “soda” OR “cola” OR “coke” OR “carbonated” OR non-alcohol* OR nonalcohol* OR “soft”) OR KW(“fizzy” OR “sugar” OR “sugary” OR “sugars” OR “sweetened” OR “soda” OR “cola” OR “coke” OR “carbonated” OR non-alcohol* OR nonalcohol* OR “soft”)

9 S7 AND S8

10 S7 NOT S9

11 (S3 AND S6) NOT S10

ABI

Dates: 22 April 2021
Years: Any
Language: Any

Source type: Books, Conference Papers & Proceedings, Dissertations & Theses, Reports, Scholarly Journals, Working Papers
Document type: Article, Book, Book Chapter, Case Study, Conference, Conference Paper, Conference Proceeding, Dissertation/Thesis, Reference Document, Review, Technical Report
1 MAINSUBJECT.EXACT("State taxes" OR "VAT" OR "GST" OR "Alternative minimum tax" OR "Ad valorem taxes" OR "Environmental tax" OR "Excise taxes" OR "Excise taxes")
2 TI(“tax” OR “taxes” OR “taxation” OR tariff* OR “duty” OR “duties” OR “excise” OR “excises” OR “levy” OR “levies”) OR AB(“tax” OR “taxes” OR “taxation” OR tariff* OR “duty” OR “duties” OR “excise” OR “excises” OR “levy” OR “levies”)
3 S1 OR S2
4 MAINSUBJECT.EXACT("Mineral water" OR "Sports drinks" OR "Tea" OR "Beverages" OR "Nonalcoholic beverages" OR "Soft drinks" OR "Milk" OR "Fruit juices" OR "Coffee" OR "Bottled water" OR "Vegetable juices" OR "Plant-based beverages" OR "Energy drinks")
5 TI(“drink” OR “drinks” OR beverage* OR “fizzy” OR “sugar” OR “sugary” OR “sugars” OR “sweetened” OR “soda” OR “cola” OR “coke” OR “carbonated”) OR AB(“drink” OR “drinks” OR beverage* OR “fizzy” OR “sugar” OR “sugary” OR “sugars” OR “sweetened” OR “soda” OR “cola” OR “coke” OR “carbonated”)
6 S4 OR S5
7 TI(alcohol*) OR AB (alcohol*)
8 TI(“fizzy” OR “sugar” OR “sugary” OR “sugars” OR “sweetened” OR “soda” OR “cola” OR “coke” OR “carbonated” OR non-alcohol* OR nonalcohol* OR “soft”) OR AB(“fizzy” OR “sugar” OR “sugary” OR “sugars” OR “sweetened” OR “soda” OR “cola” OR “coke” OR “carbonated” OR non-alcohol* OR nonalcohol* OR “soft”)
9 S7 AND S8
10 S7 NOT S9
11 (S3 AND S6) NOT S10
Source type: Scholarly Journals OR Working Papers OR Dissertations & Theses OR Conference Papers & Proceedings
NOT (Feature AND News AND Government & Official Document AND Evidence Based Healthcare AND Editorial)

Google

Conduct multiple searches, with filetype:pdf. Try using Publish or Perish to point to Google and generate a .ris file w/ PDFs
First 10 pages of each search:

1. sugar sweetened beverage tax filetype:pdf
2. soda tax filetype:pdf
3. sweet drinks tax filetype:pdf

Date searched: April 26, 2021
n=245 (some were duplicates, so not 300).

Study records

Records will be de-duplicated using Endnote, followed by a manual review, and finally by a de-duplication process within Covidence.

All de-duplicated records will be reviewed at the title/abstract and full text screening levels by two reviewers (MA and a second reviewer).

Highlights will be used in Covidence corresponding to the search strategy to aid in screening.

If title/abstract information was not available within Covidence, a Google search will be used to locate the relevant title/abstract (or a translation, if relevant).

At the title/abstract stage, we will take an inclusive approach (e.g. if there is not explicit reference to SSB taxation, but to a broader strategy which is likely to include SSB taxation, the record will be included for full text screening). For example, a title/abstract which refers to the 'NCD Best Buys' and diet-related prevention policies would be included for full text screening since the 'NCD Best Buys' report includes SSB taxation as one of several diet-related policies.

A record which mentions SSB taxation in the discussion or conclusion would still be included, and may contribute to the 'theory development' stage.

Each record will be reviewed in duplicate at both the title/abstract and full text levels, and disagreements resolved through discussion with a third reviewer.

Data extraction Stage 1: Assigning records to 'bins'

Initial data extraction will focus on assigning records to 'bins.' Initially, two reviewers will complete Stage 1 extraction on a sample of 5% of included records. Disagreements will be discussed and the extraction form may be revised for clarity. A second 5% sample may be completed if substantial changes are made. Subsequently, MA will complete the remainder of Stage 1 extraction.

We will identify two main types of studies:

1. Primary studies
    a. Empirical evaluation studies of an SSB tax or hypothetical SSB tax
        i. quantitative
        ii. qualitive (including policy process studies of failed SSB taxes)
    b. Simulation/modelling studies of an SSB tax or hypothetical SSB tax
    c. Experimental studies
2. Other studies
    a. Theory/conceptual studies
    b. Review studies
    c. Protocol studies

Initial data extraction form:

1. Study type
    a. Empirical-quantitative
    b. Empirical-qualitative

c. Empirical-mixed methods
d. Simulation/modelling
e. Experimental
f. Theory/conceptual
g. Review
h. Protocol

For study types a-e:

2. SSB stage
    a. Evidence to about a new or hypothesized SSB tax (e.g. public support of a proposed tax, experimental evidence in a virtual supermarket)
    b. Policy process around an SSB tax
    c. Evaluation of an SSB tax
    d. Evidence about an existing SSB tax (not evaluative, e.g. newspaper discourse around a tax)

For study types f-h:

3. Richness of material
    a. High (e.g. a review focused entirely on SSB taxation that covers many outcomes)
    b. Medium (e.g. a review focused on SSB taxation and only one or two outcomes; or a review of many different types of health taxes)
    c. Low (e.g. a study that mentions SSB taxation in the discussion/conclusion)

For all study types:

4. Setting: _________________ (e.g. country, city/country, or global)
5. Funding source: _______________________________
6. CoI statement: _________________________________
7. Funding and CoI seem free of industry influence:
    a. Yes
    b. No
    c. Unclear
    d. Not applicable

Data extraction Stage 2: Developing complexity-informed theory

MA will prioritize the theory/conceptual and review papers by richness, setting (e.g. global first), and year published (most recent first), and review these records in order.

We will use an Excel template to extract theoretical material from these sources. To facilitate development as a causal loop diagram, we will use a template which has previously been used to extract data from a review towards the development of a causal loop diagram.

For each included relationship, we will extract data on:

- Cause
- Effect
- Polarity [+/-] of relationship

- Delayed process [y/n] (e.g. is there a 5+ year lag between the cause and effect?)
- Excerpt [to provide context for the hypothesized causal relationship]

We are interested in capturing the breadth of theoretical relationships around SSB taxation, rather than in quantifying the number of conceptual sources that, for example, suggest price change or revenue generation are impacts of SSB taxation. Thus, we will only extract data on novel theoretical concepts for each source, unless additional nuance is added. The first several records may provide the majority of relationships, with subsequent records providing fewer novel hypothesized relationships.

Determinants or impacts which are conceptually similar will be combined.

Every relationship (represented as a unique row within the database) will be independently reviewed by a second member of the study team. Disagreements will be resolved through discussion with the whole study team.

Our focus is on the hypothesized relationships that are most proximal to SSB taxation. Thus, although we may include distal relationships (e.g. diabetes-> increased health care costs), we will not focus on these nor summarize the specific mechanisms underpinning these more distal relationships.

We will only extract data on causal statements, not descriptive ones (e.g. a descriptive statement, even with a strong implication such as "Notably, SSB taxes have also been enacted by numerous small Pacific Island nations that have some of the highest prevalences of obesity in the world" would not be used as the basis for a causal hypothesis). We will also not extract data on causal explanations for study limitations, or about other interventions (e.g taxes on sugar or salt content, instead of SSB taxes). These are all pragmatic decisions intended to help focus this stage.

We will translate the relationships into a preliminary CLD, and review the CLD and Excel database with a working group comprised of SSB taxation experts.

Data extraction Stage 3: Mapping the evidence to the complexity-informed theory

Next, we will extract data from the primary studies. We will use the list of determinants/impacts identified from the conceptual review (Stage 2) and assess which of these factors were assessed in each included study. If included studies evaluated determinants/factors not identified in the conceptual review, these categories will be added.

For each included source, we will extract data on:

1. Are any additional theoretical concepts put forward not previously included? If so, these will be added to the Excel database described above as ‘novel theoretical data.’ These data are likely in the introduction/discussion sections.
2. Which aspects of theory are tested/modelled in this study?

We will identify the factors which are empirically assessed (regardless of whether an effect is found or not) in these studies.

We will not extract effect sizes for each determinant or impact. Instead, we are interested in assessing the range of determinants and impacts which have been empirically evaluated thus far and whether there is evidence of a relationship (or absence of a relationship), regardless of effect size.

The results from this mapping review (e.g. the frequency of each setting-factor combination) will be summarized in a heat map in which each row corresponds to a unique SSB-tax setting (e.g. a country or other jurisdiction with an SSB tax).

Supplementary Information Text 3: Preferred Reporting Items for Systematic reviews and Meta-Analyses extension for Scoping Reviews (PRISMA-ScR) Checklist

SECTION	ITEM	PRISMA-ScR CHECKLIST ITEM	REPORTED ON PAGE #
**TITLE**			
Title	1	Identify the report as a scoping review.	Yes Page 1
**ABSTRACT**			
Structured summary	2	Provide a structured summary that includes (as applicable): background, objectives, eligibility criteria, sources of evidence, charting methods, results, and conclusions that relate to the review questions and objectives.	See abstract page 1
**INTRODUCTION**			
Rationale	3	Describe the rationale for the review in the context of what is already known. Explain why the review questions/objectives lend themselves to a scoping review approach.	Yes – page 2
Objectives	4	Provide an explicit statement of the questions and objectives being addressed with reference to their key elements (e.g., population or participants, concepts, and context) or other relevant key elements used to conceptualize the review questions and/or objectives.	Yes – final paragraph of Introduction, page 2
**METHODS**			
Protocol and registration	5	Indicate whether a review protocol exists; state if and where it can be accessed (e.g., a Web address); and if available, provide registration information, including the registration number.	Yes, final section of Methods, Page 9
Eligibility criteria	6	Specify characteristics of the sources of evidence used as eligibility criteria (e.g., years considered, language, and publication status), and provide a rationale.	Yes- page 8

Information sources*	7	Describe all information sources in the search (e.g., databases with dates of coverage and contact with authors to identify additional sources), as well as the date the most recent search was executed.	Yes page 7 and supplementary information
Search	8	Present the full electronic search strategy for at least 1 database, including any limits used, such that it could be repeated.	Yes; all search strategies presented in Supplementary Information Text
Selection of sources of evidence†	9	State the process for selecting sources of evidence (i.e., screening and eligibility) included in the scoping review.	Yes page 7
Data charting process‡	10	Describe the methods of charting data from the included sources of evidence (e.g., calibrated forms or forms that have been tested by the team before their use, and whether data charting was done independently or in duplicate) and any processes for obtaining and confirming data from investigators.	Yes- pages 7-8
Data items	11	List and define all variables for which data were sought and any assumptions and simplifications made.	Yes – page 8
Critical appraisal of individual sources of evidence§	12	If done, provide a rationale for conducting a critical appraisal of included sources of evidence; describe the methods used and how this information was used in any data synthesis (if appropriate).	It was beyond the scope of this review to include a critical appraisal.
Synthesis of results	13	Describe the methods of handling and summarizing the data that were charted.	Yes – page 9
**RESULTS**			
Selection of sources of evidence	14	Give numbers of sources of evidence screened, assessed for eligibility, and included in the review, with reasons for exclusions at each stage, ideally using a flow diagram.	Yes, see Figure 1.
Characteristics of sources of evidence	15	For each source of evidence, present characteristics for which data were charted and provide the citations.	See Supplementary Information and Data Source File

Critical appraisal within sources of evidence	16	If done, present data on critical appraisal of included sources of evidence (see item 12).	It was beyond the scope of this review to include a critical appraisal.
Results of individual sources of evidence	17	For each included source of evidence, present the relevant data that were charted that relate to the review questions and objectives.	See Data Source File
Synthesis of results	18	Summarize and/or present the charting results as they relate to the review questions and objectives.	See Figures 2-5
**DISCUSSION**			
Summary of evidence	19	Summarize the main results (including an overview of concepts, themes, and types of evidence available), link to the review questions and objectives, and consider the relevance to key groups.	Yes, pages 5-6
Limitations	20	Discuss the limitations of the scoping review process.	Yes page 6
Conclusions	21	Provide a general interpretation of the results with respect to the review questions and objectives, as well as potential implications and/or next steps.	Yes pages 6-7
**FUNDING**			
Funding	22	Describe sources of funding for the included sources of evidence, as well as sources of funding for the scoping review. Describe the role of the funders of the scoping review.	Yes – page 10

JBI = Joanna Briggs Institute; PRISMA-ScR = Preferred Reporting Items for Systematic reviews and Meta-Analyses extension for Scoping Reviews.

* Where *sources of evidence* (see second footnote) are compiled from, such as bibliographic databases, social media platforms, and Web sites.

† A more inclusive/heterogeneous term used to account for the different types of evidence or data sources (e.g., quantitative and/or qualitative research, expert opinion, and policy documents) that may be eligible in a scoping review as opposed to only studies. This is not to be confused with *information sources* (see first footnote).

‡ The frameworks by Arksey and O'Malley (6) and Levac and colleagues (7) and the JBI guidance (4, 5) refer to the process of data extraction in a scoping review as data charting.

§ The process of systematically examining research evidence to assess its validity, results, and relevance before using it to inform a decision. This term is used for items 12 and 19 instead of "risk of bias" (which is more applicable to systematic reviews of interventions) to include and acknowledge the various sources of evidence that may be used in a scoping review (e.g., quantitative and/or qualitative research, expert opinion, and policy document)

From: Tricco AC, Lillie E, Zarin W, O'Brien KK, Colquhoun H, Levac D, et al. PRISMA Extension for Scoping Reviews (PRISMAScR): Checklist and Explanation. Ann Intern Med. 2018;169:467–473. doi: 10.7326/M18-0850.

References

1. Sterman, J. D. System Dynamics: Systems Thinking and Modeling for a Complex World. in *Engineering Systems Division Internal Symposium, Massachusetts Institute of Technology* 31 (2002).
2. Best, A., Clarke, P., Leischow, S. & Trochim, W. *Greater Than the Sum: Systems Thinking in Tobacco Control*. https://cancercontrol.cancer.gov/sites/default/files/2020-06/m18_complete.pdf (2007).
3. Ross, H. L. Law, Science, and Accidents: The British Road Safety Act of 1967. *The Journal of Legal Studies* **2**, 1–78 (1973).
4. McGill, E. *et al.* Evaluation of public health interventions from a complex systems perspective: a research methods review. *Social Science & Medicine* 113697 (2021) doi:10.1016/j.socscimed.2021.113697.
5. Sterman, J. *Business Dynamics: Systems Thinking and Modeling for a Complex World*. (Irwin/McGraw-Hill, 2000).
6. *Developing and evaluating complex interventions*. https://mrc.ukri.org/documents/pdf/complex-interventions-guidance/ (2019).
7. Panter, J., Guell, C., Prins, R. & Ogilvie, D. Physical activity and the environment: conceptual review and framework for intervention research. *International Journal of Behavioral Nutrition and Physical Activity* **14**, 156 (2017).
8. Grant, M. J. & Booth, A. A typology of reviews: an analysis of 14 review types and associated methodologies: A typology of reviews, *Maria J. Grant & Andrew Booth*. *Health Information & Libraries Journal* **26**, 91–108 (2009).
9. Gough, D., Kiwan, D., Suttcliffe, K., Simpson, D. & Houghton, N. A systematic map and synthesis review of the effectiveness of personal development planning for improving student learning. (2006).
